# Heterogeneity in the treatment of bloodstream infections identified from antibiotic exposure mapping

**DOI:** 10.1002/pds.4761

**Published:** 2019-03-27

**Authors:** Aisling R. Caffrey, Zachary R. Babcock, Vrishali V. Lopes, Tristan T. Timbrook, Kerry L. LaPlante

**Affiliations:** ^1^ Infectious Diseases Research Program and Center of Innovation in Long‐term Services and Supports Veterans Affairs Medical Center Providence Rhode Island USA; ^2^ College of Pharmacy University of Rhode Island Kingston Rhode Island USA; ^3^ Department of Health Services, Policy and Practice Brown University School of Public Health Providence Rhode Island USA; ^4^ Department of Pharmacy University of Utah Health Salt Lake City Utah USA

**Keywords:** antibiotics, bacteremia, exposure mapping, pharmacoepidemiology, treatment patterns, Staphylococcus aureus

## Abstract

**Purpose:**

As changes in antibiotic therapy are common, intent‐to‐treat and definitive therapy exposure definitions in infectious disease clinical trials and observational studies may not accurately reflect all antibiotics received over the course of the infection. Therefore, we sought to describe changes in antibiotic therapy and unique treatment patterns among patients with bacteremia.

**Methods:**

We conducted a retrospective cohort study of hospitalizations from Veterans Affairs (VA) Medical Centers (January 2002‐September 2015) and community hospitals (de‐identified Optum Clinformatics DataMart with matched Premier Hospital data; October 2009‐March 2013). In the VA population, antibiotic exposures were mapped from the culture collection date among those with positive *Staphylococcus aureus* cultures. In the Optum‐Premier population, exposures were mapped from the admission date among those with a primary diagnosis of bacteremia.

**Results:**

Our study included 50 467 bacteremia admissions, with only 14% of admissions having the same treatment pattern as another admission. For every 100 bacteremia admissions, 89 had changes in antibiotic therapy. For every 100 bacteremia admissions with changes in therapy, 95 had unique antibiotic treatment patterns. These findings were consistent in both populations, over time, and among different facilities within study populations. The median time to first therapy change was 2 days after initial therapy, with a median of three changes.

**Conclusions:**

Changes in antibiotic therapy for bloodstream infections were nearly universal regardless of hospital setting. Based on our findings, common antibiotic exposure definitions of intent‐to‐treat and definitive therapy would misclassify exposure in 86% of admissions, which highlights the need for better operational definitions of exposure in infectious diseases research.

**KEY POINTS**

- Among more than 50 000 bacteremia admissions, antibiotic treatment was highly heterogeneous in two distinct study populations, over time, and among different facilities within each study population.
- Changes in antibiotic therapy occurred in 89% of admissions (median of three changes over course of treatment), with 95% having unique treatment patterns.
- Common antibiotic exposure definitions of intent‐to‐treat and definitive therapy would misclassify exposure in 86% of the study population, which highlights the need for better operational definitions of exposure in infectious diseases research.

## 1 INTRODUCTION

Serious infections, such as drug‐resistant bloodstream infections, have become increasingly complicated to treat.^1^, ^2^ Initial treatment decisions are often made without knowledge of the infecting organism(s) and in the absence of a confirmed source of infection.^1^ Management of these patients is challenging as bloodstream infections are associated with high mortality rates, and evidence suggests that immediate treatment with appropriate therapies can significantly improve survival.^1^, ^3^, ^4^ However, further complicating these decisions are the lack of real‐world evidence regarding the most effective and safe treatment approaches, including which antibiotics to use and their duration of use.^5^, ^6^

The complex treatment regimens used to treat infectious diseases, which consist of relatively short exposure periods and multiple changes in therapy, create great difficulty in accurately defining antibiotic exposures for the evaluation of clinical success. Often, patients are started on empiric broad‐spectrum antibiotics for a suspected infection.^1^, ^7^ This therapy may be continued for 1 to 5 days, depending on whether rapid diagnostics are available, when clinical culture results are received, and if/when infectious disease clinicians become involved. Once culture results are available, patients should be switched to targeted or definitive therapy based on the infecting organism and related susceptibilities.^8^ However, some may be continued on broad‐spectrum therapy. Other changes may be made in preparation for hospital discharge, where intravenous therapy is switched to oral therapy, or cases of insufficient clinical response. Combination therapy regimens further complicate efforts to measure antimicrobial exposures due to potential additive or synergistic effects. As the spectrum of common antibiotic regimens and real‐world patterns of treatment for serious infections have yet to be described, the objective of this study was to map all antibiotic exposures for those with bacteremia. As treatment practices may vary by health system, we included both Veterans Affairs (VA) Medical Centers (VAMCs) and community hospitals in our study. Further, treatment practices will vary by causative organism; therefore, we included both an organism‐specific cohort and a disease‐state cohort.

## 2 METHODS

We utilized two data sources for this retrospective cohort study, the national VA databases and a de‐identified Clinformatics DataMart (OptumInsight, Eden Prairie, Minnesota) with matched Premier Hospital data. In the VA population, we included hospital inpatients with positive blood cultures for *Staphylococcus aureus* (methicillin‐susceptible [MSSA] and methicillin‐resistant [MRSA]) between 1 January 2002 and 30 September 2015. In the Optum‐Premier population, patients were included if they were hospitalized between 1 October 2009 and 31 March 2013 with a primary diagnosis of bacteremia or septicemia (International Classification of Diseases, Ninth Revision, Clinical Modification [ICD‐9‐CM] codes 003.1, 020.2, 022.3, 036.2, 038.0, 038.1, 038.10‐038.12, 038.19, 038.2, 038.3, 038.40‐038.44, 038.49, 038.8, 038.9, 054.5, 449, 771.81, 995.91, 995.92, 790.7) by any causative organism.^9^ As microbiological culture results were not available in the Optum‐Premier data, three additional inclusion criteria were applied: (1) at least 6 months of baseline eligibility without a primary diagnosis of bacteremia or septicemia, (2) initiation of antibiotic(s) on the day of admission or the day after admission, and (3) at least two consecutive days of antibiotic therapy within the first 3 days of the admission. For both cohorts, only adults (age ≥ 18 y) were selected for inclusion and multiple admissions were included if the subsequent admission date was more than 30 days from the previous discharge date.

In the VA population, the index date was the culture collection date. In the Optum‐Premier population, the index date was the admission date as culture data were not available. Daily antibiotic exposures were mapped from the culture collection date until discharge. For admissions with greater than 30 days between the index and discharge dates, only the first 30 days were included. VA pharmacy data included barcode medication administration records and pharmacy dispensings. Optum‐Premier pharmacy data were ascertained from inpatient hospital charge records.

Changes in therapy were identified and summed per patient. The median time to first change was calculated for those with changes in therapy. Treatment patterns were defined from both antibiotic exposures and duration of exposure. Dose changes and changes from intravenous to oral forms of the same antibiotic were not considered changes in therapy. Unique treatment patterns were defined as those where a single admission had a specific pattern of antibiotic exposures and durations and no other admission shared the same pattern. Nonunique treatment patterns were defined as those where multiple admissions had the same pattern of antibiotic exposures and durations. For example, the following was a treatment pattern shared by several admissions, vancomycin and piperacillin‐tazobactam for 4 days, with a switch to vancomycin alone for 3 days, while the following was a unique treatment pattern observed in a single admission, vancomycin and piperacillin‐tazobactam for 4 days, with a switch to vancomycin alone for 2 days, with a switch to nafcillin for 3 days.

Trends in treatment pattern heterogeneity over time were assessed with joinpoint regression (Joinpoint Regression Program version 4.6.0.0; National Cancer Institute, Bethesda, Maryland). Further, we conducted two sensitivity analyses, one in which we revised the exposure mapping definition to account for holds of 1 day, where one or more therapies were held for a single day and resumed the following day, and wider dosing frequencies (ie, every 48 h). As such, holds, extended dosing, and 1‐day gaps were not counted as changes in therapy in this sensitivity analysis. In the second sensitivity analysis, unique patterns were assessed only for antibiotic exposures and did not account for days of therapy that allowed us to assess treatment strategies rather than day‐specific patterns.

As heterogeneity of antibiotic treatments for bacteremia may vary by length of stay, mortality, infection source, and causative organism, we conducted several stratified analyses. For example, we would expect greater homogeneity in treatment patterns during shorter hospital stays, as well as with specific infection sources or causative organisms, whereas heterogeneity may be greater among those who die during the admission. We therefore assessed unique antibiotic treatment patterns stratified by length of stay, osteomyelitis (VA population only and diagnosis code present during the admission), inpatient mortality, and methicillin susceptibility of *S aureus* (VA population only).^7^ Length of stay was stratified at 7 days and also by median length of stay in each setting (Table 1).

**Table 1:** Antibiotic treatment patterns

	Veterans Affairs Medical Centers	Optum‐Premier
	n = 47,584 Admissions	n = 2,883 Admissions
Age, median (IQR)	64 (57‐75)	58 (48‐65)
Male, no. (%)	46 509 (97.7%)	1476 (51.2%)
Charlson comorbidity index, median (IQR)	3 (2‐5)	2 (0‐3)
Length of stay, d, median (IQR)	11 (6‐20)	5 (3‐9)
Inpatient mortality, no. (%)	8504 (17.9%)	191 (6.6%)
Change in therapy		
Number with change, no. (%)	42 220 (88.7%)	2437 (84.5%)
Day of change, median (IQR)	2 (1‐3)	2 (2‐3)
Number of changes, median (IQR)	3 (2‐6)	2 (1‐4)
Unique patterns, no. (%)	39 825 (94.3%)	2375 (97.5%)
No change in therapy		
Number without change, no. (%)	5364 (11.3%)	446 (15.5%)
Unique patterns, no. (%)	1218 (22.7%)	179 (40.1%)
Overall heterogeneity	41 043 (86.3%)	2554 (88.6%)
	43 597 (86.4%)	

Abbreviations: IQR, interquartile range; no., number. Unique patterns indicate those where a single admission had a specific pattern of antibiotic exposures and durations and no other admission shared the same pattern.

## 3 RESULTS

We identified 50 467 bacteremia admissions (47 584 VA, 2883 Optum‐Premier; Table 1). The VA population was older (median age 64 versus 58 y) and mostly male (97.7% versus 51.2%) compared with the Optum‐Premier population. Further, the VA population had a greater comorbidity burden (median Charlson comorbidity index 3 versus 2), longer length of stay (median 11 versus 5 d), and higher inpatient mortality rate (17.9% versus 6.6%).

**Figure 1:**
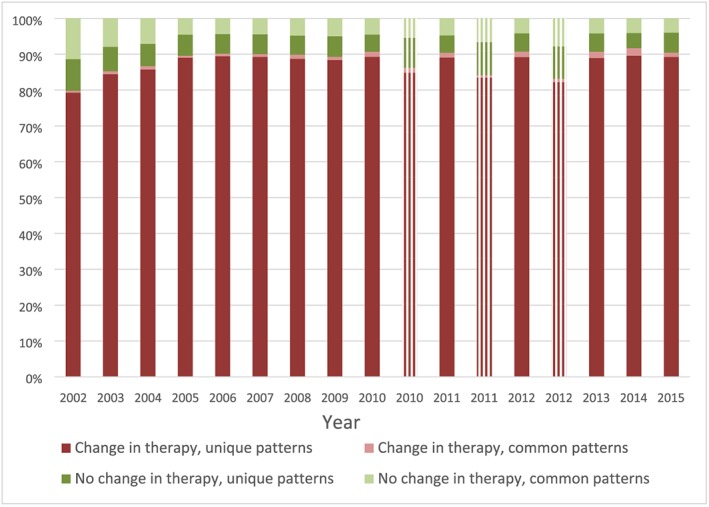
Antibiotic treatment patterns by year. Veterans Affairs (VA) Medical Centers are represented with solid bars, and Optum‐Premier are represented with lined bars. Unique patterns indicate those where a single admission had a specific pattern of antibiotic exposures and durations and no other admission shared the same pattern. Common patterns indicate those where multiple admissions had the same pattern of antibiotic exposures and durations. In the VA population, a significant increase in heterogeneity was observed between 2002 and 2005 (annual percent change 2.3%, *P* < 0.05); however, heterogeneity remained stable thereafter (*P* = 0.3) [Colour figure can be viewed at wileyonlinelibrary.com]

**Figure 2:**
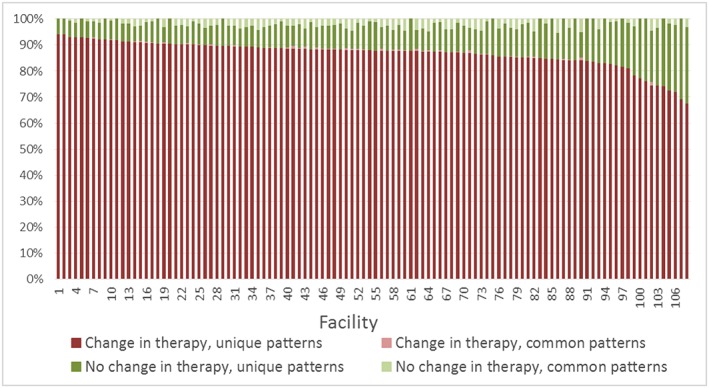
Antibiotic treatment patterns by facility, Veterans Affairs Medical Centers. Unique patterns indicate those where a single admission had a specific pattern of antibiotic exposures and durations and no other admission shared the same pattern. Common patterns indicate those where multiple admissions had the same pattern of antibiotic exposures and durations. Excluding 14 facilities with less than 20 admissions [Colour figure can be viewed at wileyonlinelibrary.com]

**Figure 3:**
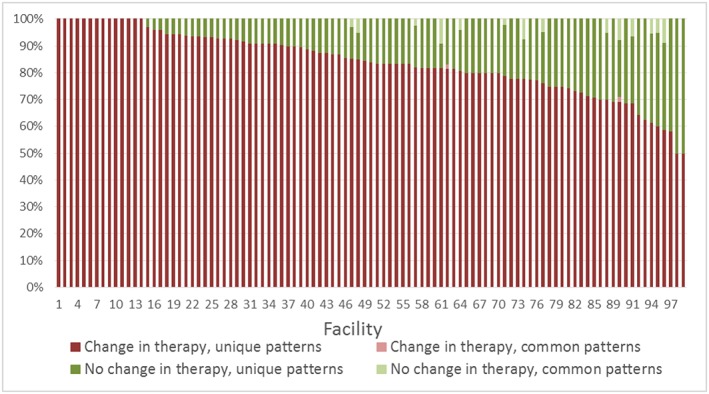
Antibiotic treatment patterns by facility, Optum‐Premier. Unique patterns indicate those where a single admission had a specific pattern of antibiotic exposures and durations and no other admission shared the same pattern. Common patterns indicate those where multiple admissions had the same pattern of antibiotic exposures and durations. Excluding 269 facilities with less than 10 admissions [Colour figure can be viewed at wileyonlinelibrary.com]

Rates of therapy changes were similar in both populations as shown in Table 1 (88.7% VA, 84.5% Optum‐Premier). The median time to first therapy change was 2 days after the initial therapy in both the VA and Optum‐Premier populations. The median number of changes was three for the VA population and two for the Optum‐Premier population. The number of unique change patterns per the number of admissions with changes in therapy was also similar in both populations (94.3% VA, 97.5% Optum‐Premier). Heterogeneous (unique) treatment patterns were observed in 86.3% of the VA population and 88.6% of the Optum‐Premier population (overall 86.4%). These findings were consistent over time (Figure 1) and between facilities (Figures 2 and 3). Although heterogeneity increased significantly between 2002 and 2005 (annual percent change 2.3%, *P* < 0.05) in the VA population, it remained stable thereafter (*P* = 0.3).

**Figure 4:**
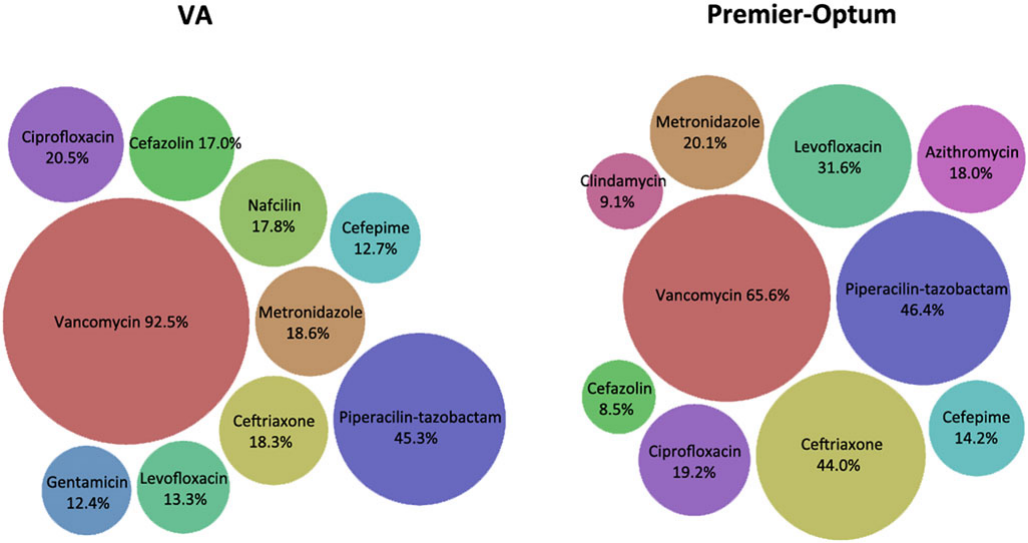
Changes in therapy: utilization of specific antibiotics within treatment patterns. Percentages indicate number of treatment patterns that included that specific antibiotic per number of bacteremia admissions (Veterans Affairs [VA] Medical Centers n = 42 220, Optum‐Premier n = 2437) [Colour figure can be viewed at wileyonlinelibrary.com]

Among admissions with changes in therapy, vancomycin and piperacillin‐tazobactam were the most commonly identified antibiotics within treatment patterns for both study populations (Figure 4).^10^ Some variation was observed between settings in the percentage of patterns with a specific antibiotic, such as with vancomycin (92.5% VA, 65.6% Optum‐Premier), which was expected since the VA study population included patients with *S aureus* bacteremia while the Optum‐Premier population included those with diagnoses of bacteremia caused by any organism. Alternatively, utilization was similar for other antibiotics, such as with piperacillin‐tazobactam (45.3% VA, 46.4% Optum‐Premier) and ciprofloxacin (20.5% VA, 19.2% Optum‐Premier).

**Figure 5:**
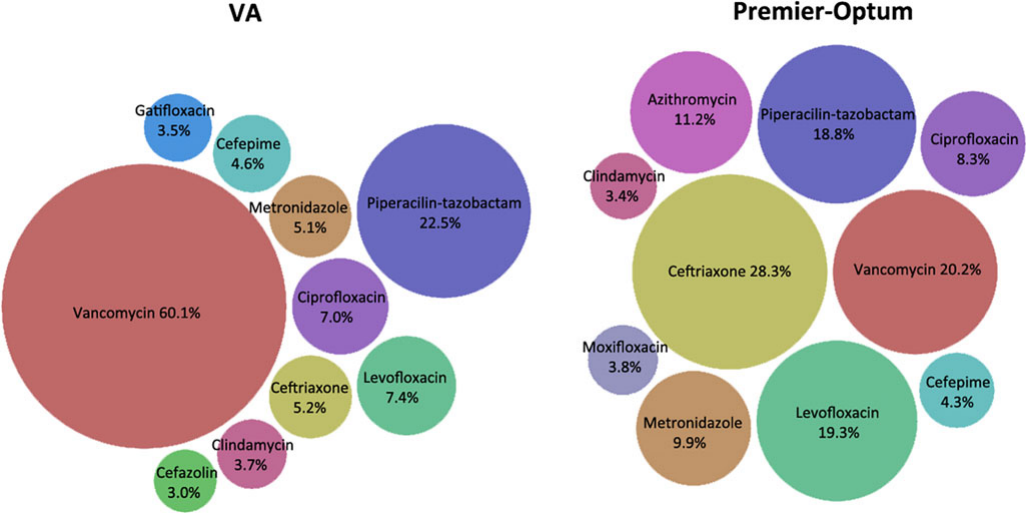
Without changes in therapy: utilization of specific antibiotics within treatment patterns. Percentages indicate number of treatment patterns that included that specific antibiotic per number of bacteremia admissions (Veterans Affairs [VA] Medical Centers n = 5364, Premier‐Optum n = 446) [Colour figure can be viewed at wileyonlinelibrary.com]

Greater variation in unique treatment patterns between the study populations was observed for admissions without changes in therapy (22.7% VA, 40.1% Optum‐Premier). Among admissions without changes in therapy, vancomycin was the most commonly identified antibiotic within treatment patterns in the VA population (60.1%), while ceftriaxone (28.3%) was the most common antibiotic in the Optum‐Premier population (Figure 5).^10^ The second most common antibiotic was piperacillin‐tazobactam in the VA population (22.5%) and vancomycin (20.2%) in the Optum‐Premier population.

Common treatment patterns among admissions both with and without changes in therapy differed between the study populations. The top 250 treatment patterns (sorted by count, then alphabetically) for each population, with at least four observations per pattern, can be found in Tables S1.1 to S1.4. In the VA population, combination therapy with piperacillin‐tazobactam and vancomycin for various durations, with a change to either vancomycin monotherapy or piperacillin‐tazobactam monotherapy for various durations, was among the common patterns observed (Table S1.1). Monotherapy, with either vancomycin or piperacillin‐tazobactam, along with combination therapy of the two, was the common treatment observed among those without changes in therapy (Table S1.2). In Optum‐Premier, the top 10 patterns included seven different antibiotics (Table S1.3). Monotherapy, with either vancomycin or levofloxacin, was the common treatment observed among those without changes in therapy (Table S1.4). Duration of antibiotic therapy was 4 days or less among the top five patterns in each population (Tables S1.1‐S1.4). While changes in therapy were slightly lower when accounting for holds, extended dosing, and 1‐day gaps (Table S1.5; 87.5% VA, 84.5% Optum‐Premier), proportions of unique patterns were similar (93.9% VA, 97.0% Optum‐Premier).

In stratified analyses by length of stay, heterogeneity was lower in shorter hospital stays, although it still exceeded 70% in shorter stays and was greater than 90% in longer stays (Tables S1.6‐S1.7; ≤ median length of stay as noted in Table 1, 78.9% VA, 80.6% Optum Premier, > median length of stay as noted in Table 1, 95.7% VA, 98.9% Optum‐Premier; ≤ 7 days length of stay, 73.4% VA, 84.0% Optum‐Premier, > 7 days length of stay, 93.9% VA, 99.8% Optum‐Premier). Heterogeneity was high in patients with a concomitant diagnosis of osteomyelitis (Table S1.8; 94.5%). In the VA population, heterogeneity was similar in stratified analyses by inpatient mortality (Table S1.9; inpatient mortality 84.8%, survival 87.8%); however in the Optum‐Premier population, heterogeneity was higher among those who died during the admission than among those who survived (95.8% and 88.4%). Heterogeneity was also similar by methicillin susceptibility (Table S1.10; 88.7% MSSA, 84.8% MRSA).

In sensitivity analyses evaluating unique antibiotic treatment strategies without length of therapy, among admissions with changes in therapy, the proportion with unique patterns remained high (Table S2.1; 78.0% VA, 87.7% Optum‐Premier; overall heterogeneity 70.3% VA, 76.8% Optum‐Premier). In the VA and Optum‐Premier populations, combination therapy with piperacillin‐tazobactam and vancomycin with a change to vancomycin monotherapy occurred in 1.4% and 0.49% of admissions, respectively (Tables S2.2 and S2.4). Alternatively, common treatment patterns among admissions without changes in therapy were similar between the study populations (Tables S2.3 and S2.5): vancomycin monotherapy (29.1% VA, 4.7% Optum‐Premier), combination therapy with piperacillin‐tazobactam and vancomycin (11.0% VA, 5.4% Optum‐Premier), and piperacillin‐tazobactam monotherapy (4.7% VA, 10.1% Optum‐Premier). Heterogeneity was again higher in longer hospital stays (Tables S2.7‐S2.8) and among those who died during the admission in the Optum‐Premier population (Table S2.9; 92.7%).

## 4 DISCUSSION

To our knowledge, this is the first study to describe the full spectrum of real‐world treatment patterns for bacteremia in two national cohorts. Exposure mapping has traditionally been applied in adherence research, specifically to long‐term treatments for chronic diseases, such as diabetes or hypercholesterolemia.^11^ By creating drug treatment maps for each patient on each day of the observation period, adherence research is able to calculate exposure measures such as proportion of days covered, medication possession ratio, and gaps in therapy.^11^, ^12^ Using this approach, we were able to identify changes in antibiotic therapy and unique patterns of antibiotic treatments. For every 100 bacteremia admissions, 89 had changes in antibiotic therapy, and for every 100 bacteremia admissions with changes in therapy, 95 had different antibiotic treatment patterns. Heterogeneous treatment patterns were identified in 86 of every 100 bacteremia admissions.

Interestingly, the observed heterogeneity in treatment patterns persisted over time was consistent in the clinically distinct study populations and was similar among different facilities within each study population. With the release of MRSA treatment guidelines in 2011, we expected a change in unique patterns; however, heterogeneity remained stable in subsequent years.^7^ When considering unique patterns collectively among those with and without changes in therapy, variability exceeded 90% even among the facilities with the lowest percentage of unique patterns.

We did expect to observe greater homogeneity in treatment patterns in the VA study population for three reasons: (1) We included admissions with a specific infection type (bacteremia) caused by a specific organism (*S aureus*) that was confirmed from positive blood cultures, (2) these were admissions from a closed health care system, and (3) the study population tends to be more homogenous in terms of patient characteristics. We also expected to observe greater heterogeneity in the Optum‐Premier population since we included admissions with diagnoses of bacteremia caused by any organism. While heterogeneity could be interpreted as higher than expected in the VA and perhaps lower than expected in Optum‐Premier, heterogeneity was high in both groups (86.3% VA, 88.6% Optum‐Premier).

Total duration of antibiotic exposure was low among the common patterns, indicating that these common patterns were observed among patients who were discharged soon after culture/admission. We did not assess postdischarge outpatient antibiotic treatments as we were specifically interested in inpatient treatment patterns. Had we included the full duration of treatment, which would have been a minimum of 2 weeks for *S aureus* bacteremia and may include postdischarge changes to oral or outpatient parenteral antibiotic therapy to facilitate hospital discharge, heterogeneity likely would have been even greater.^7^ Further, in assessing treatment strategies that did not account for days of therapy, heterogeneity still exceeded 70%. Interestingly, heterogeneity remained high in shorter hospital stays, exceeding 70%, by inpatient mortality and methicillin susceptibility, exceeding 80% in all groups, and in a single infection source (osteomyelitis), exceeding 90%. Although we expected heterogeneity to be somewhat higher with MRSA than MSSA, MSSA treatment heterogeneity was actually slightly higher (88.7% MSSA, 84.8% MRSA).

In infectious diseases research, antibiotic exposure definitions tend to be overly broad and may not accurately reflect the full spectrum of treatment. In both clinical trials and observational studies, these oversimplified definitions lead to misclassification by considering certain periods of treatment as ignorable. Such ignorable periods therefore assume equivalence of efficacy/effectiveness between all other antibiotics utilized in the treatment of that infection and for any duration. For example, intent‐to‐treat does not take into account concomitant or subsequent exposures after randomization. As such, in clinical trials that allow for adjunctive therapy and changes in therapy, postrandomization antibiotics are treated as ignorable.^5^, ^6^, ^13^, ^14^, ^15^, ^16^, ^17^, ^18^, ^19^ Additionally, antibiotic therapy prior to randomization may also be treated as ignorable, and despite randomization, exposures may differ significantly between study populations.^5^, ^14^, ^16^, ^17^, ^18^, ^19^ It is difficult to assess the impact of therapy prior to randomization, adjunctive therapy, or changes in therapy without explicit reporting in trial results and sensitivity analyses assessing the impact of these other antibiotic exposures.

In observational research, definitive therapy is a common exposure definition, which identifies treatment after a certain time point, for example, after culture results are available.^20^, ^21^, ^22^, ^23^ Periods treated as ignorable may include either empiric or definitive therapy, or both, where previous and subsequent treatments are not assessed, not reported, and/or not controlled for.^20^, ^21^, ^22^, ^23^ This approach assumes that any other antibiotics received, for any duration, are equal in terms of beneficial and harmful effects. Operational definitions of definitive therapy also differ by study, where periods of empiric and definitive therapy may vary between patients^20^, ^22^ or be set equal for all patients.^21^, ^24^ A solution to this misclassification has been to include those without changes in therapy, treated with monotherapy or simple combinations. However, as our study demonstrates, these patterns are uncommon in real‐world clinical practice.

As re‐evaluation of antibiotic therapy is a core element of antimicrobial stewardship, we expected to see high rates of therapy changes.^8^ However, we did not expect such extensive heterogeneity in real‐world treatment patterns. It is unclear whether the variability in treatment approaches for the same infection represents the forefront of individualized medicine, where each host‐organism relationship is unique and requires a distinct approach to treatment, or, alternatively, whether the variability results from a lack of quality evidence supporting specific treatment regimens, particularly specific antibiotics for specific durations.

To avoid inaccurate exposure definitions in infectious disease research, there are several solutions for handling these traditionally ignored exposure periods. First, clinical trials could be designed so that randomization accounts for empiric therapy. Second, sensitivity analyses could be conducted in clinical trials and observational studies that control for differences in empiric therapy prior to randomization/definitive therapy, as well as differences in antibiotic exposures after randomization/definitive therapy. Third, time‐varying methods could be used to account for both time‐varying antibiotic exposures and time‐varying outcomes. Fourth, highly specific operational exposure definitions could be used in observational research, where study populations are restricted to patients with similar exposure patterns as identified from daily exposure mapping. The addition of any of these approaches would provide a more accurate description of the relationship between treatment and the clinical outcomes being assessed.

## 5 LIMITATIONS

The main limitation of the study was the use of diagnosis codes to identify bacteremia in the Optum‐Premier population. Treatment patterns will vary by causative organism; however, microbiology data were not available from the data source. Bacteremia diagnosis codes have demonstrated varying sensitivity and positive predictive value for positive blood cultures.^25^ However, we observed similar heterogeneity in prescribing patterns and related implications on characterizing treatment effects between our culture‐confirmed bacteremia population and the population identified from diagnosis codes. Second, we did not assess antibiotic dose. Had we included dose, we expect that variability in antibiotic patterns would be close to 100%. Third, we did not exclude patients with concomitant infections, and therefore, some of the antibiotic exposures may have been targeting other infecting organisms. Fourth, we did not evaluate heterogeneity in treatment patterns by patient characteristics, such as age and comorbidity burden. However, since heterogeneity was high in the overall populations, it would have also been within patient subgroups. Lastly, we did not assess postdischarge antibiotic treatments. Therefore, if a switch to oral therapy occurred prior to discharge, it was captured in the pattern. However, if the prescription was dispensed after the discharge date, it was not captured as part of the pattern. The generalizability of this study is limited to the two national cohorts included, patients admitted to VAMCs and patients from the Optum Clinformatics database admitted to Premier community hospitals.

## 6 CONCLUSIONS

Changes in antibiotic therapy for bloodstream infections were nearly universal in both VAMCs and community hospitals. Common treatment approaches, consisting of homogenous (nonunique) treatment patterns, were used in only 14% of bacteremia admissions. This heterogeneity in antibiotic treatment patterns was consistent over time and was similar among different facilities within each study population. Our findings highlight the challenges of evidence‐based research for the treatment of infectious diseases. Since so few patients receive the same regimen, true head‐to‐head comparisons may not be possible due to small numbers and therefore rely on overly broad definitions. As antibiotic exposure definitions are unlikely to be as accurate as previously assumed, noninferiority, superiority, comparative effectiveness, and comparative safety studies in infectious diseases should be interpreted with caution.

## ETHICS STATEMENT

This study was approved by the Institutional Review Board and Research and Development Committee of the Providence VAMC and approved as exempt by the University of Rhode Island's Institutional Review Board.

## CONFLICT OF INTEREST

Aisling Caffrey has received research funding from Pfizer, Merck (Cubist), and The Medicines Company. Zachary Babcock and Vrishali Lopes have no conflicts to disclose. Tristan Timbrook has received honoraria for speaking and/or consulting from BioFire Diagnostics, GenMark Diagnostics, and Roche Diagnostics. Kerry LaPlante has received research funding or acted as a scientific advisor for Allergan, Bard, Merck (Cubist), Pfizer, and The Medicines Company.

## AUTHOR CONTRIBUTIONS

*Conception, study design, and protocol development*: A.R.C. and K.L.L. *Data generation*: A.R.C., Z.R.B., V.V.L., and T.T.T. *Analysis and/or interpretation of data*: A.R.C., Z.R.B., V.V.L., T.T.T., and K.L.L. *Preparation and critical revision of the final manuscript*: A.R.C., Z.R.B., V.V.L., T.T.T., and K.L.L.

## Supporting information

Table S1.1 Top 250 treament patterns: with change in therapy, Veterans Affairs Medical Centers
Table S1.2 Top 250 treament patterns: without change in therapy, Veterans Affairs Medical Centers
Table S1.3 Top 250 treament patterns: with change in therapy, Optum‐Premier
Table S1.4 Top 250 treament patterns: without change in therapy, Optum‐Premier
Table S1.5 Antibiotic treatment patterns, accounting for holds, extended dosing, and one day gaps
Table S1.6 Antibiotic treatment patterns by median length of stay
Table S1.7 Antibiotic treatment patterns by 7‐day length of stay
Table S1.8 Antibiotic treatment patterns, Staphylococcus aureus with osteomyelitis diagnosis
Table S1.9 Antibiotic treatment patterns by inpatient mortality
Table S1.10 Antibiotic treatment patterns, methicillin‐susceptible and resistant Staphylococcus aureus

Table S2.1 Antibiotic treatment patterns
Table S2.2 Top 250 treament patterns: with change in therapy, Veterans Affairs Medical Centers
Table S2.3 Top 250 treament patterns: without change in therapy, Veterans Affairs Medical Centers
Table S2.4 Top 250 treament patterns: with change in therapy, Optum‐Premier
Table S2.5 Top 250 treament patterns: without change in therapy, Optum‐Premier
Table S2.6 Antibiotic treatment patterns, accounting for holds, extended dosing, and one day gaps
Table S2.7 Antibiotic treatment patterns by median length of stay
Table S2.8 Antibiotic treatment patterns by 7‐day length of stay
Table S2.9Antibiotic treatment patterns by inpatient mortality
